# Health‐related quality of life in patients with heart failure eligible for treatment with sacubitril–valsartan

**DOI:** 10.1002/nop2.420

**Published:** 2019-11-19

**Authors:** Lena Holmlund, Margareta Brännström, Krister Lindmark, Camilla Sandberg, Karin Hellström Ängerud

**Affiliations:** ^1^ Department of Nursing Umeå University Umeå Sweden; ^2^ Department of Public Health and Clinical Medicine Umeå University Umeå Sweden

**Keywords:** health‐related quality of life, heart failure, self‐care, self‐efficacy

## Abstract

**Aim:**

To describe and compare self‐reported health‐related quality of life between younger and older patients with severe heart failure eligible for treatment with sacubitril–valsartan and to explore the association between health‐related quality of life and age, NYHA classification, systolic blood pressure and NT‐proBNP level.

**Design:**

Cross‐sectional study.

**Methods:**

A total of 59 patients, eligible for treatment with sacubitril–valsartan were consecutively included and divided into a younger (≤75 years) and older group (>75 years). Health‐related quality of life was assessed using the Kansas City Cardiomyopathy Questionnaire and the EuroQol 5‐dimensions. Data were collected between June 2016 and January 2018. The STROBE checklist was used.

**Results:**

There were no differences in overall health‐related quality of life between the age groups. The older patients reported lower scores in two domains measured with the Kansas City Cardiomyopathy Questionnaire, namely self‐efficacy (67.0 *SD* 22.1 vs. 78.8 *SD* 19.7) and physical limitation (75.6 *SD* 19.0 vs. 86.3 *SD* 14.4). Higher NYHA class was independently associated with lower Kansas City Cardiomyopathy Questionnaire Overall Summary Score.

## 1 INTRODUCTION

In developed countries, 1%–2% of the adult population are affected by heart failure (HF) and among people >70 years of age the prevalence is more than 10%. However, the number of undiagnosed cases is presumed to be high. The syndrome can rarely be cured, but drugs and various forms of treatment have been developed and implemented in the past 30 years resulting in increased survival and reduced hospitalization in patients with HF (Ponikowski et al., 2016). Health‐related quality of life (HRQoL) in patient with HF is reported low and is associated with a large number of symptoms such as fatigue, shortness of breath, pain, dry mouth and drowsiness. Even with optimal treatment, the clinical manifestations of HF differ from patient to patient, and the overall disease burden has an impact on all aspects of quality of life and is comparable to other severe chronic diseases such as Parkinson's disease (Calvert, Freemantle, & Cleland, 2005). Different opinions exist in the field regarding the impact of ageing on HRQoL in patients with HF. To better understand the role of ageing and HRQoL in patients with HF, this study provides a discussion of this phenomenon.

## 2 BACKGROUND

Pharmacological treatment of HF with reduced ejection fraction is, according to recommendations in European guidelines, angiotensin‐converting enzyme inhibitors (ACE‐I) with the addition of a beta blocker. Mineralocorticoid/aldosterone receptor antagonist (MRA) is recommended for patients who have symptoms despite the treatment of the two primary drugs (Ponikowski et al., 2016). This regimen has for decades been the standard pharmacological treatment of HF. Recently, the PARADIGM‐HF‐study was discontinued prematurely as it has shown beneficial effects with sacubitril–valsartan compared with ACE‐I in terms of increased survival (McMurray et al., 2014b) and improved HRQoL (Chandra et al., 2018; Lewis et al., 2017). Therefore, sacubitril–valsartan is now recommended in the ESC guidelines for patients with HF with a reduced ejection fraction and who are symptomatic despite optimal medication (Ponikowski et al., 2016). Swedish Association of Local Authorities and Regions have decided that the drug shall be introduced nationwide (Regions, 2016). The introduction might be complex (Farmakis, Bistola, Karavidas, & Parissis, 2016), because the population with HF is ageing, with most over 75 years and with statistically significant comorbidities (Zarrinkoub et al., 2013). In many clinical trials, older and frail patients often are excluded. The real‐world population who meet the criteria for treatment with sacubitril–valsartan according to ESC guidelines is significantly older than the selected participants in the PARADIGM‐HF study (Norberg, Bergdahl, & Lindmark, 2018) and can, therefore, have other needs of support when starting a new medication.

Patients considered for treatment with sacubitril–valsartan will access care at different HF clinics for distribution of the drug. It is important that healthcare professionals who will meet and follow‐up with these patients know how patients with severe HF perceive their HRQoL. A factor important for quality of life is self‐care self‐efficacy (Tsay & Healstead, 2002), and self‐efficacy is (in turn) important for the patient to manage HF self‐care (Buck et al., 2015). self‐efficacy is a concept developed by Albert Bandura and is a supporting part of his social cognitive theory. The concept refers to the individual's confidence in his or her ability to cope with and act in a specific situation. It is not about a general belief in their own ability, but it is a belief in their own ability to cope with a specific situation (Bandura, 1977).

Health‐related quality of life in patients with HF has been studied and described extensively (Calvert et al., 2005; Friedman, 2003; Masoudi et al., 2004), but to our knowledge there have been few studies describing HRQoL in patients with HF who meet the criteria for treatment with sacubitril–valsartan according to the main PARADIGM‐HF study criteria (McMurray et al., 2014b). Since the HF population largely consists of older people, it is important to find out if there are differences in estimated HRQoL between younger and older patients with HF and therefore different needs of support. We hypothesize that HRQoL differs between younger and older patients with HF. Hopefully, knowledge from the present study will contribute to health professionals being more prepared to meet the needs that might arise around these patients, such as compliance with medication, self‐care and symptom relief. In the long run, this might lead to symptom improvement, which in turn might improve patients' ability to cope with daily life and ultimately increase HRQoL in patients with severe HF.

The aim of the present study was to describe and compare self‐reported HRQoL between younger and older patients with severe HF who are eligible for treatment with sacubitril–valsartan and to explore the association between HRQoL and age, NYHA class, systolic blood pressure and NT‐proBNP levels. The research questions addressed in this paper are: are there differences in self‐reported HRQoL between younger and older patients with severe HF who are eligible for treatment with sacubitril–valsartan? Are there associations between HRQoL and age, NYHA class, systolic blood pressure and NT‐proBNP levels?

## 3 METHODS

### 3.1 Study design and setting

The present study had a cross‐sectional design with self‐reported data. A cross‐sectional design with questionnaires was chosen because it was considered appropriate for the purpose of this study. It was a part of a wide‐ranging project in a hospital healthcare setting aiming to investigate epidemiology, prevention, genesis, reasons for treatment and testing of methods for introducing new treatments in patients with HF. Details about the main study have been described previously (Norberg et al., 2018). The study took place at a university hospital in Sweden, and the research context was an HF clinic. The STROBE checklist was applied (Appendix S1).

### 3.2 Participants

All patients with a diagnosis of HF who lived in the catchment area of the university hospital in Sweden between January 2010–March 2016 were documented in a local database at the clinic. At least one registered contact with the internal medicine or heart clinic at the hospital was mandatory for inclusion. In the local database, 1924 patients with HF were identified, and following the PARADIGM‐HF criteria (McMurray et al., 2014b), 95 patients met the criteria for treatment with sacubitril–valsartan. This procedure was previously described in detail (Norberg et al., 2018). After manual follow‐up of the medical records, 19 patients with a changed condition were excluded and 76 patients met the criteria for treatment and were invited to the HF outpatient clinic. The aim was to include all who were initiated on sacubitril–valsartan but finally, 59 patients were consecutively included between June 2016–January 2018.

### 3.3 Data collection

Health‐related quality of life was assessed using the EuroQol‐5D (EQ‐5D) and the Kansas City Cardiomyopathy Questionnaire (KCCQ). The questionnaires were available in Swedish and translated into several languages, making it easier to compare the results with other studies using the same instruments. Background data in the survey consisted of age, gender, marital status, lodging and education. Data relating to blood pressure, NT‐proBNP and NYHA classification were obtained from the patient's records from the visit.

### 3.4 EuroQol‐5D

The EQ‐5D defines health in the five dimensions of mobility, self‐care, usual activities, pain/discomfort and anxiety/depression. Each question has three levels for the respondent to classify their health, including no problems, some problems and extreme problems (“EuroQol–a new facility for the measurement of health‐related quality of life,” 1990). The merged data can be converted into a single summary index, the EQ‐5D_index_ (Rabin & de Charro, 2001), where perfect health gives an EQ‐5D_index_ value of 1 and the worst possible condition or death has an EQ‐5D_index_ value of 0. The British index was adopted as the reference in the absence of a Swedish index (Drummond, 2005). The EQ‐5D self‐report also consists of a vertical, visual analogue scale (VAS 0–100) where 0 is the worst imaginable state and 100 is the best imaginable state (EuroQol, 1990). The EQ‐5D is a validated tool (Ellis, Eagle, Kline‐Rogers, & Erickson, 2005) that is reliable, easy to use and responsive to change (Hurst, Kind, Ruta, Hunter, & Stubbings, 1997).

### 3.5 Kansas City Cardiomyopathy Questionnaire

Kansas City Cardiomyopathy Questionnaire is a disease‐specific questionnaire that measures and quantifies self‐reported health status in patients with HF (Green, Porter, Bresnahan, & Spertus, 2000). The items are grouped into six domains and two summary scores. The six domains are labelled physical limitation, Total Symptom Score, Symptom Change, self‐efficacy, Social Interference and quality of life. The Clinical Summary Score (CSS) is a summary of physical limitations and Total Symptom Score, and the Overall Summary Score (OSS) is a summary of the CSS, quality of life and Social Interference. The scale scores transform to a scale of 0–100, where lower scores indicate worse perceived health status and higher scores indicate better perceived health status. The Swedish version of the KCCQ is a valid and reliable tool for use with the chronic HF population in Sweden, with Cronbach's alpha for the KCCQ scales varying from 0.67–0.92 (Patel, Ekman, Spertus, Wasserman, & Persson, 2008). In this study, Cronbach's alpha for the domains and summary scores were physical limitation = 0.81, Total Symptom Score = 0.84, self‐efficacy = 0.65, Social Interference = 0.83, quality of life = 0.86, CSS = 0.90 and OSS = 0.94.

### 3.6 Statistical analysis

Statistical analysis was performed using Statistical Packages for the Social Sciences version 24 (SPSS, IBM). Categorical data are presented as numbers and percentages, and the continuous data are presented as means and standard deviation (*SD*). The patients were divided into two groups, younger (≤75 years) and older (>75 years), based on the median age as the cut‐off. The median was chosen to divide groups into younger and older, due to skewness in the group. Chi‐square test or Fischer's exact test for categorical variables was used to compare proportions between groups, and continuous variables were compared using the Mann–Whitney *U* test since the sample size was small. Cronbach's alpha was used to assess the reliability of the KCCQ domains and summary scores. A multiple logistic regression was used to investigate the associations between lower and higher KCCQ‐OSS (the dependent variable) and age group, systolic blood pressure, NYHA class and NT‐proBNP level (the independent variables). The independent variables included in the logistic regression were chosen because they can indicate deterioration of HF and deemed to be important for HRQoL. The median, 84.2, was used as cut‐off for the dependent variable (OSS) because of skewness in the group. Odds ratios (ORs) with 95% confidence intervals (CIs) were used to present the results. The results were considered statistically significant if *p* < .05.

### 3.7 Ethics

The study was approved by the regional ethical review board, Dnr 2016‐233‐32M (Addition to 2015‐419‐31 M). Oral and written informed consent was obtained from all participants, and the investigation followed the principles outlined in the Declaration of Helsinki (World Medical Association, 2013).

## 4 RESULTS

Background characteristics for the whole group and the groups of younger and older participants are shown in Table 1. The group was predominately male but there was no statistically significant difference between the groups regarding gender. The older group of patients had lower levels of education compared with the younger patients (Table 1), and systolic blood pressure was higher in the group of older patients (Table 1).

**Table 1:** Background characteristics

	Total *N* = 59	Younger *N* = 30	Older *N* = 29	*p*
Sex				
Female	5 (8.5)	2 (6.7)	3 (10.3)	.671
Male	54 (91.5)	28 (93.3)	26 (89.7)	
Age	72.6 ± 11.2	64.8 ± 10.5	80.6 ± 3.7	**<.001**
Age, range	31–90	31–75	76–90	
Accommodation				
Cohabiting	44 (74.6)	22 (37.3)	22(37.3)	.824
Living alone	15 (25.4)	8 (13.6)	7 (11.9)	
Education				
Elementary school	22 (37.3)	6 (20.0)	16 (55.2)	**.019**
Secondary school	24 (40.7)	16 (53.3)	8 (27.6)	
University/college	13 (22)	8 (26.7)	5 (17.2)	
NT‐proBNP, pg/ml	2,244	2,030	2,669	.095
Blood pressure				
Systolic, mmHg	121 ± 17	116 ± 13	126 ± 19	**.041**
Diastolic, mmHg	71 ± 11	69 ± 12	72 ± 10	.354
NYHA classification				
Unknown	11 (18.6)	7 (23.3)	4 (13.8)	.331
NYHA 2	13 (22.0)	8 (26.7)	5 (17.2)	
NYHA 3	35 (59.3)	15 (50.0)	20 (69.0)	

Note

Data are presented as *N* (%) or mean ± standard deviation. Bold figures denote statistically significant *p*‐values.

Abbreviations: NT‐proBNP; N‐terminal pro‐B‐type natriuretic peptide, NYHA; New York Heart Association.

### 4.1 EQ5D_index_ and EQ‐VAS

No differences were found between the two groups regarding HRQoL assessed with the EQ‐5D_index_ (0.76 *SD* 0.27 vs. 0.75 *SD* 0.20 for the older group vs. the younger group) or for the EQ‐VAS (62.3 *SD* 17.5 vs. 68.23 *SD* 15.7, respectively) (Table 2).

**Table 2:** The results of the analysis of the quality of life measurements, measured with EQ‐5D and the Kansas City Cardiomyopathy Questionnaire

	Total	Younger	Older	*p*
	Mean ± *SD* *N* = 59	Mean ± *SD* *N* = 30	Mean ± *SD* *N* = 29	
EQ5D‐index	0.76 ± 0.23	0.75 ± 0.20	0.76 ± 0.27	.462
EQ5D‐VAS	65.38 ± 16.8	68.23 ± 15.7	62.3 ± 17.5	.218
KCCQ score				
Physical limitation	80.4 ± 17.8	86.3 ± 14.4	75.6 ± 19.0	**.023**
Total symptom score	80.1 ± 18.6	80.9 ± 17.5	79.2 ± 20.0	.860
Symptom stability score	49.1 ± 13.3	50.0 ± 14.7	48.1 ± 11.9	.942
Social limitation score	80.0 ± 21.1	82.4 ± 19.0	77.5 ± 23.1	.574
Self‐efficacy score	73.1 ± 21.6	78.8 ± 19.7	67.0 ± 22.1	**.038**
QoL‐score	74.3 ± 23.5	75.8 ± 21.1	72.6 ± 26.1	.694
OSS	78.7 ± 17.6	82.6 ± 13.1	75.4 ± 20.3	.354
CSS	81.2 ± 16.5	85.8 ± 12.3	77.2 ± 18.6	.089

Note

The data are presented as mean ± standard deviation. Bold figures denote statistically significant *p*‐values.

Abbreviations: CSS, Clinical Summary Score; KCCQ, Kansas City Cardiomyopathy Questionnaire; OSS, Overall Summary Score; QoL, quality of life.

### 4.2 Kansas City Cardiomyopathy Questionnaire

The entire group of patients reported a mean OSS of 78.7 (*SD* 17.6) and a mean CSS of 81.2 *SD* 16.5. In the comparison of the groups of older and younger patients, the older patients had lower scores in both these summary scores, but these differences were not statistically significant (Table 2). In all domains of the KCCQ, there were no differences between younger and older patients, except for self‐efficacy where the older group had significantly lower scores compared with the younger group (67.0 *SD* 22.1 vs. 78.8 *SD* 19.7, *p* = .038) and for physical limitation where the older group also reported lower scores than the younger group (75.6 *SD* 19.0 vs. 86.3 *SD* 14.4, *p = *.023) (Table 2).

### 4.3 Factors associated with KCCQ overall summary score

The univariate logistic regression analysis showed that NYHA classification III was associated with lower KCCQ‐OSS (*p* = .016) (Table 3). Age, systolic blood pressure and NT‐proBNP level were not associated with lower KCCQ‐OSS. The multivariate analyses showed that patients with higher NYHA classification were independently associated with lower KCCQ‐OSS (*p* = .010, Table 3).

**Table 3:** Univariate and multivariate logistic regression of factors associated with impaired KCCQ Overall Summary Score

	Univariate analysis			Multivariate analysis		
	OR	95% CI	*p*	OR	95% Cl	*p*
NYHA class	14.73	1.64–132.64	**.016**	20.56	2.05–206.40	**.010**
Age	1.19	0.37–3.81	.767	0.97	0.20–4.71	.974
Systolic blood pressure	0.99	0.95–1.02	.414	0.97	0.93–1.02	.226
NT‐proBNP	1.00	1.00–1.00	.923	1.00	1.00–1.00	.784

Note

Bold figures denote statistically significant *p*‐values.

Abbreviations: KCCQ, Kansas City Cardiomyopathy Questionnaire; NYHA class, New York Heart Association classification; NT‐proBNP, N‐terminal pro‐B‐type natriuretic peptide.

## 5 DISCUSSION

The present study showed that patients with HF who were eligible for treatment with sacubitril–valsartan rated their HRQoL high on the group level. This was consistent in the subgroup analysis of younger and older patients. In the analysis of the dimensions of the KCCQ, the older patients estimated both self‐efficacy and physical limitation lower than the younger patients. The logistic regression analysis showed that higher NYHA classification was a factor that was negatively associated with HRQoL, and the worse the function the lower the reported HRQoL. Age, systolic blood pressure and NT‐proBNP level were not associated with HRQoL.

An important finding in the present study was that the older patients rated their self‐efficacy significantly lower compared with younger patients. To our knowledge, this has not been reported previously, but in the maintenance of self‐care, self‐efficacy seems to be important for improving HRQoL (Buck et al., 2015). According to Bandura (1977), there are four sources of self‐efficacy: performance achievements, vicarious experience, verbal persuasions and emotional arousal. Performance achievement: this source of information is based on personal experience and is therefore considered to be particularly influential. Vicarious experience is, for example, to see another individual with the same disease or condition, manage a specific task. Verbal persuasion means that the person is encouraged or persuaded to cope with a particular task or situation. Emotional arousal is the individual's mental and physical experience of the situation and with negative feelings about the situation, and self‐efficacy tends to decrease (Bandura, 1977). The fact that self‐efficacy was scored lower among the older patients might provide information on how healthcare professionals should respond to patients with HF based on their condition, both in terms of education about the disease and about self‐care behaviour. But this is a complex phenomenon, and there are many factors that need to be taken into account. Confidence in HF self‐care affects HRQoL (Buck et al., 2012), and self‐care for patients with HF involves, among other things, knowledge of the disease and the performance of activities to prevent deterioration in one's condition. The older patients in the present study had a lower education level than the younger patients, and Dennison et al. (2011) concluded that both the older people and those with lower education have a lower degree of health literacy. Health literacy is a person's capability to read, understand, obtain and use health‐related information to maintain and promote good health (World Health Organization, 2016). Dennison et al. (2011) suggest that knowledge about the disease and confidence in self‐care is important among patients with HF, especially the older people and those with less than a high school education. Further, patients with HF often suffer comorbidity and cognitive difficulties are also common, especially in older people (Ponikowski et al., 2016). Moreover, comorbidities and cognitive difficulties are likely to negatively affect compliance with medication and adherence to self‐care. Because old age is associated with lower health literacy (Baker, Gazmararian, Sudano, & Patterson, 2000; Dennison et al., 2011) and low health literacy is in turn related to a poorer health outcome (Baker et al., 2000), it is important to improve self‐efficacy by tailoring interventions that seek to improve HRQoL.

The present study showed no difference between younger and older patients in overall HRQoL, which is in contrast to previous reports (Masoudi et al., 2004; Moser et al., 2013; Zambroski, Moser, Bhat, & Ziegler, 2005). The fact that younger patients to a greater extent than older patients with HF report their quality of life to be lower is notable. This might be due to that fact that older patients to a greater extent accept their condition and have fewer expectations of themselves because a decrease in physical function is an expected part of normal ageing (Moser et al., 2013). In the PARADIGM‐HF study (McMurray et al., 2014a), the patients reported their HRQoL to be lower than the patients in this study when comparing OSS (73 vs. 79, respectively) and they were on average more than eight years younger than the patients in this study (64 vs. 72.6 years, respectively). In the PARADIGM‐HF study, McMurray et al. (2014a) compared their results to other major randomized multicentre studies that have approximately comparable parameters in terms of age and HRQoL, that is younger patients and low self‐rated HRQoL. This finding, that the patients in the PARADIGM‐HF study reported lower scores for HRQoL than the patients in our study, might be due to the fact that they were younger. After all, there were no differences in overall HRQoL between the age groups in our study, but this was a rather small population. Regarding self‐reported HRQoL assessed with the EQ‐5D_index_, older people reported almost the same HRQoL as the younger patients, 0.76 compared with 0.75. A study on HRQoL in a general population, measured with EQ‐5D_index_, showed that older people rated their HRQoL lower than younger people and those aged 60–79 years scored 0.78 on the EQ‐5D_index_ and those aged 80–88 years scored 0.74 (Burstrom, Johannesson, & Diderichsen, 2001). These results are in contrast to the present study, although their data originated from a general population. In addition, comparisons in HRQoL between diseases and socio‐economic groups in their study showed that there were large variations (Burstrom et al., 2001).

In the present study, it was shown that poorer functioning status was associated with lower HRQoL. This finding complies with Zambroski et al. (2005) who also showed that higher NYHA classification predicts worse HRQoL. A deterioration in functional capacity in older people will significantly decrease HRQoL, while in younger patients functional deterioration does not lead to a decrease in HRQoL (Masoudi et al., 2004). This indicates the importance of making improvements both through new medication as well as improving self‐care in these patients to reduce the symptom burden, especially in older people, to prevent progression of the disease and deterioration of HRQoL. It is required that health care for people with chronic conditions has to be improved to meet the needs of an ageing population. One way is to develop and implement digital self‐care support, but it is important to be aware of that older people might have lower self‐efficacy and reduced ability to handle such digital tools. It is also recommended to enrol patients with HF in multidisciplinary management programs (Ponikowski et al., 2016). Multidisciplinary management programs might differ in content between different healthcare settings, but a common goal is to achieve a system of care that provides a structured follow‐up through optimization of medication, education, improved access to care and support (Ponikowski et al., 2016). Person‐centred care might be a good alternative to regular care because it has been shown to improve the quality of life of patients with HF (Brannstrom & Boman, 2014). A literature review showed that person‐centred care also improves self‐efficacy and self‐care as well as mental and physical status, lowers health costs, improves treatment adherence, ensures patient dignity and decreases symptom burden (Ulin, Malm, & Nygardh, 2015).

### 5.1 Methodological considerations and limitations

This study took place at a single centre and had a small sample size, which limits the statistical significance and complicates the possibility of generalization. The small sample size also risking to yield type II errors, namely false‐negative results. A cross‐sectional design was chosen because it was considered appropriate for the purpose of this study, but this might have made it difficult to see causal relationships. A strength was that both a generic and a disease‐specific instrument were used in the measurement of HRQoL. The EQ5D, EQ‐VAS and KCCQ are tools that are available in Swedish. Moreover, they are valid, reliable and relatively easy to use and understand (Ellis et al., 2005; Hurst et al., 1997; Patel et al., 2008) and are translated into several languages, making it easier to compare our results with other studies using the same instruments. The research group consisted of different professions, which was also a strength.

## 6 CONCLUSIONS

To summarize, the present study showed that there were no differences in overall HRQoL between younger and older patients with HF eligible for sacubitril–valsartan. The older patients reported lower self‐efficacy and more pronounced physical limitation compared with the younger patients. NYHA classification, but not age, systolic blood pressure or NT‐proBNP level, was associated with worse HRQoL. Person‐centred care, including targeted support in self‐care, might be one way to prevent deterioration of the disease and might strengthen self‐efficacy in older patients.

## 7 RELEVANCE TO CLINICAL PRACTICE

In the increasingly large population of older people with HF and with comorbidities such as multiple illnesses, dementia and confusion, it is important to be aware that self‐efficacy might be lower in this group. Person‐centred care might be a good alternative to regular care to improve the quality of life of patients with HF and by tailored interventions seek to improve self‐efficacy in older people. Still, more research is needed to determine how to fully implement person‐centred care in health care, but a good start is to really see the patient and not just the disease and to help the patient to increase their quality of life through education and self‐care based on the patient's condition.

## CONFLICT OF INTEREST

KL has received lecture grants from Novartis. For the remaining authors, none were declared.

## AUTHOR CONTRIBUTIONS

KHÄ and LH: Data collection. LH, KHÄ and CS: Statistical analyses. LH: Manuscript drafting. KHÄ, KL, CS and MB: Manuscript preparation and critical revision for important intellectual content. All authors have read and approved the final manuscript.
